# Cytochrome P450-catalyzed allylic oxidation of pentalenene to 1-deoxypentalenic acid in pentalenolactone biosynthesis

**DOI:** 10.1016/j.engmic.2025.100206

**Published:** 2025-04-05

**Authors:** Jing Li, Chengde Zhang, Shiwen Wu, Jiao Xue, Ke Chen, Zixin Deng, Dongqing Zhu

**Affiliations:** a The Key Laboratory of Combinatorial Biosynthesis and Drug Discovery (Ministry of Education), Wuhan University, Wuhan 430071, China; b Biomedicine Research Center, the Third Affiliated Hospital of Guangzhou Medical University, Guangzhou 510150, China

**Keywords:** *Streptomyces*, Pentalenene oxygenase, Ferredoxin, Ferredoxin reductase, Pentalenolactone

## Abstract

Pentalenolactone is a sesquiterpene antibiotic from *Streptomyces*. Its biosynthetic pathway has been elucidated, except for the oxidation of pentalen-13-al to 1-deoxypentalenic acid. In this study, we show that cytochrome P450 pentalenene oxygenase catalyzed the formation of 1-deoxypentalenic acid. Ferredoxin XNR_5179 and ferredoxin reductase XNR_4478 from *S. albus* are suitable redox proteins for pentalenene oxygenase. The biosynthetic pathway presented fills a gap in the biosynthetic pathway of pentalenolactone and provides an example of cytochrome P450 enzyme activity being affected by redox proteins.

## Graphical abstract

**Figure:**
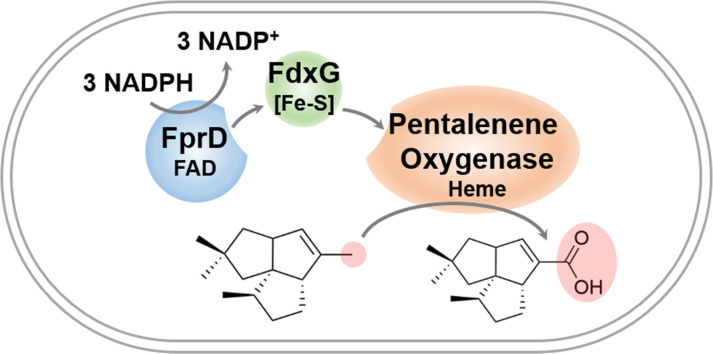

Cytochrome P450s are a family of heme-containing monooxygenases that catalyze various oxidative reactions [1]. Decades after their discovery, P450 systems have been recognized as powerful biocatalysts in synthetic biology [2,3]. Pentalenolactone (**1**) is a sesquiterpene derived from farnesyl diphosphate (FPP, **2**) (Fig. 1) [[4], [5], [6], [7]]. Pentalenene oxygenase, encoded by the cytochrome P450 gene *ptlI* from the pentalenolactone (**1**) biosynthetic gene cluster of *Streptomyces avermitilis* [8], catalyzes the conversion of pentalenene (**3**) to pentalen-13-al (**5**) via stepwise oxidation of pentalen-13-ol (**4**) [9]. An open question remains as to how aldehyde pentalen-13-al (**5**) is converted into 1-deoxypentalenic acid (**6**). It is conceivable that another enzyme within the biosynthetic gene cluster is responsible for this conversion. Subsequently, the pentalenolactone biosynthetic pathway was identified in *S. avermitilis, S. exfoliatus* and *S. arenae* [[10], [11], [12], [13], [14], [15], [16], [17], [18], [19], [20]], and no genes involved in the biosynthesis of **6** were found in these gene clusters.

**Fig. 1:**
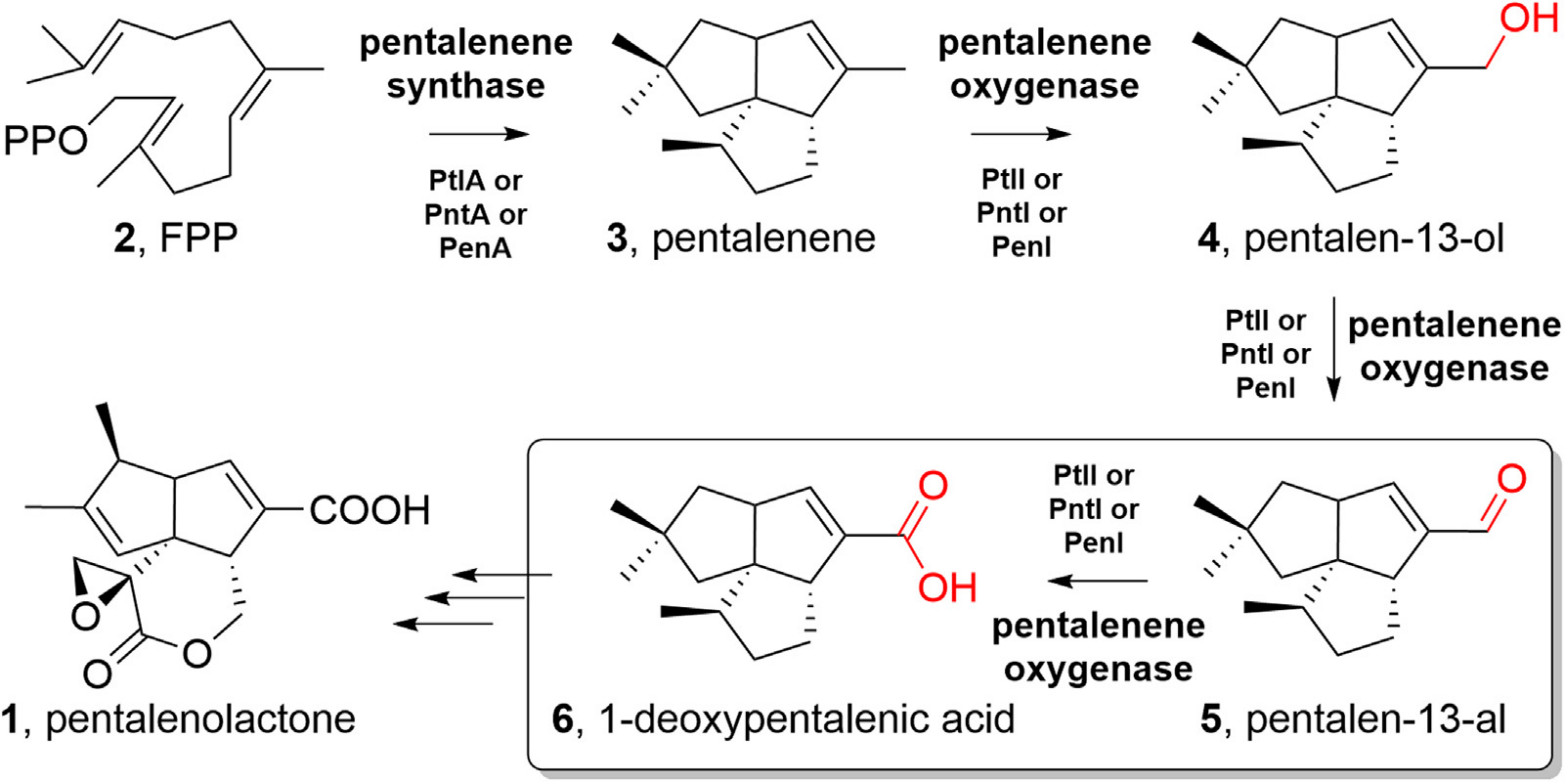
Biosynthesis of pentalenolactone (**1**).

Cytochrome P450s catalyze numerous oxygenation reactions including the three-step oxidation of a methyl group to a carboxylic acid. FscP in FR-008/candicidin biosynthesis [21], AmphN in amphotericin biosynthesis [22], NysN in nystatin biosynthesis [23], ScnG/PimG in pimaricin biosynthesis [24], and RosC in 20-carboxyrosamicin biosynthesis [25] are all type examples from bacteria. This type of three-step oxidation catalyzed by P450 also occurs during the biosynthesis of natural plant products, such as celastrol [26], ent‑kaurenoic acid [27,28], bile acid [29], artemisinic acid [30,31], and carlactonoic acid [32]. Therefore, we speculated that pentalenene oxygenase PtlI in *S. avermitilis* and its homologs PenI in *S. exfoliatus* and PntI in *S. arenae* might be responsible for the allylic oxidation of **3** to **6** under appropriate conditions (Fig. 1). In this study, we focused on the pentalenene oxygenase genes *pntI* from *S. arenae* and *penI* from *S. exfoliatus* and proved the above hypothesis using heterologous expression in *Streptomyces* and *Escherichia coli*. We also identified suitable electron transport proteins for pentalenene oxygenase that promote the catalytic efficiency of this reaction.

First, we deleted the pentalenene oxygenase gene *penI* in the pentalenolactone producer *S. exfoliatus* UC5319 to generate a Δ*penI* mutant CD2, in which an internal 1194-bp fragment (from 100 nt to 1293 nt) in *penI* was replaced by a 6-bp *Nde*I site (Fig. S1 A; all bacterial strains and plasmids used in this work were listed in Tables S1 and S2). PCR amplification with the primer pair UCF and UCR yielded a 601-bp product from the double-crossover strain *S. exfoliatus* CD2 and a 1795-bp product from the wild-type strain *S. exfoliatus* UC5319 (Fig. S1 B), all of which are listed in Table S3. Wild-type *S. exfoliatus* UC5319 and the *penI* mutant CD2 were then cultured, acidified, and extracted. The organic extracts were dried, concentrated, methylated, and analyzed using gas chromatography-mass spectrometry (GC–MS). The GC–MS results showed that the wild-type strain UC5319 produced **1** (Fig. 2A and B). The *penI* mutant CD2 did not produce any detectable **1** or accumulated **3** (Fig. 2C and D), which is the cyclization product of FPP (**2**) catalyzed by pentalenene synthase and was used as the substrate for the reaction catalyzed by recombinant PtlI *in vitro* [9]. We also deleted the *pntI* gene in another pentalenolactone producer, *S. arenae* TU469, to generate a Δ*pntI* mutant. GC–MS analysis confirmed that the *pntI* mutant generated accumulated pentalenene (**3**) and did not produce **1**, unlike the wild-type strain TU469 (not shown).

**Fig. 2:**
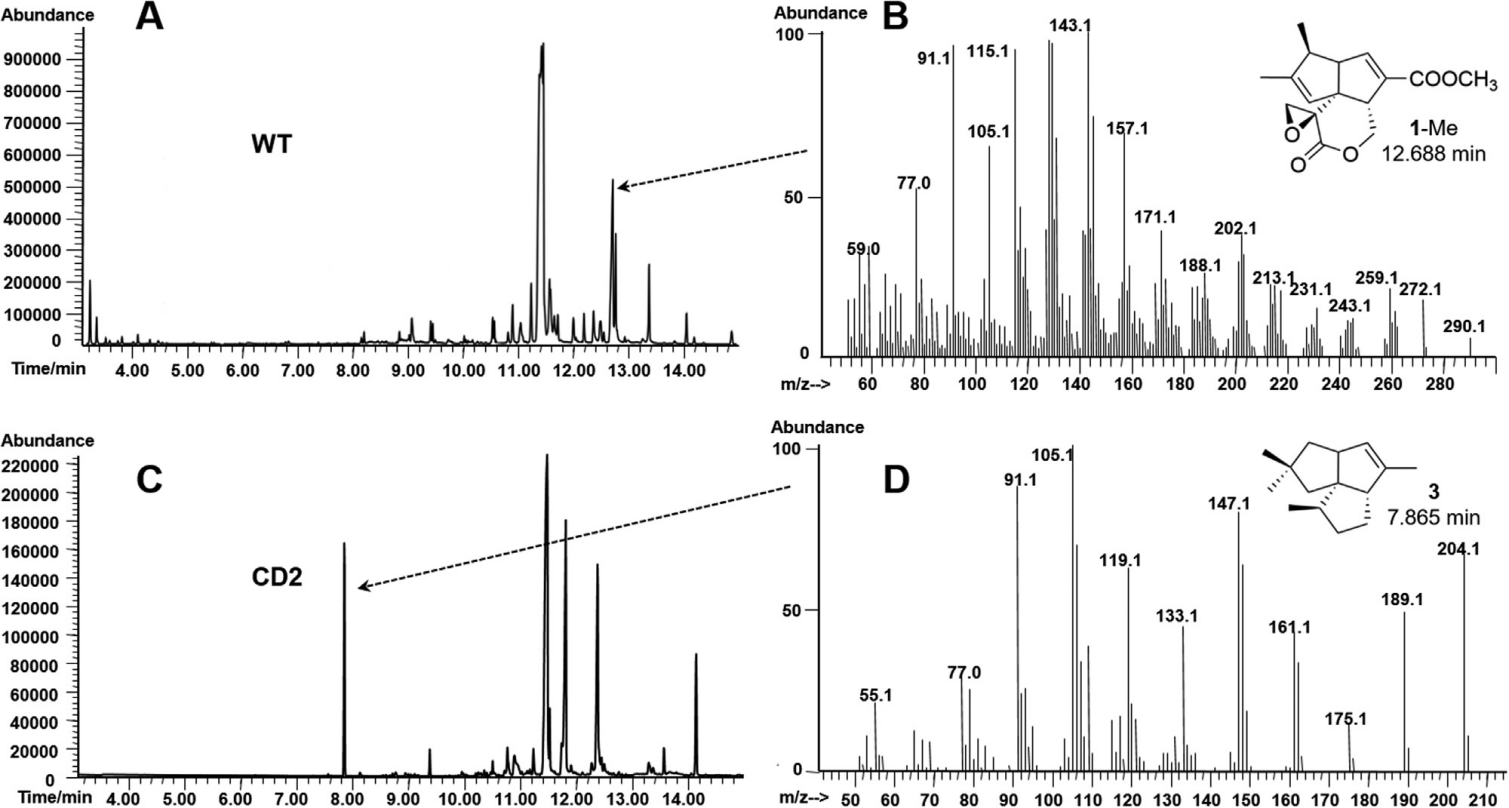
GC–MS analysis of the *S. exfoliatus penI* mutant CD2. A, Gas chromatography (GC) analysis of wild-type *S. exfoliatus* UC5319. B, Mass spectra (MS) of **1**-Me produced by the *S. exfoliatus* UC5319. C, GC analysis of the *S. exfoliatus penI* mutant CD2. D, MS of **3** produced by the *S. exfoliatus penI* mutant CD2.

Next, two *Streptomyces* strains, *S. albus* and *S. lividans*, which do not contain the pentalenolactone biosynthetic gene cluster, were selected as hosts for the heterogeneous expression of the pentalenene oxygenase gene. The DNA fragment carrying the pentalenene synthase gene *pntA* and the pentalenene oxygenase gene *pntI* was amplified from *S. arenae* TU469 and inserted into the integrative vector pIB139 carrying the constitutively strong promoter *ermE*p* to generate the plasmid pWHU1702. Plasmid pWHU1703, which contains *pntA*, was used as a control. Plasmids pWHU1702 and pWHU1703 were then transformed into *S. albus* J1074 and *S. lividans* TK24, respectively. The resulting strains were cultured, acidified, and extracted. The organic extracts were dried, concentrated, methylated, and analyzed via GC–MS. GC-MS analysis of *S. albus* revealed that the wild-type strain J1074 did not produce any intermediates of **1** (Fig. 3A) and that J1074::pWHU1703 produced **3** (Fig. 3B and C), the product of FPP catalyzed by PntA. Further, 1-deoxypentalenic acid methyl ester (**6**-Me) was detected in the culture of J1074::pWHU1702 (Fig. 3D and E). GC–MS analysis of the cultures of *S. lividans* gave the same results as *S. albus*: **6**-Me was found in the culture of TK24::pWHU1702 (Fig. 3F and G) but not in the cultures of the control strains TK24 and TK24::pWHU1703 (not shown). Next, **4** and **5** were not detected in the cultures of J1074::pWHU1702 and TK24::pWHU1702, revealing that PntI catalyzed **3** to **6** efficiently in *Streptomyces* hosts. In summary, these results indicate that pentalenene oxygenase catalyzes the allylic oxidation of **3** to **6**.

**Fig. 3:**
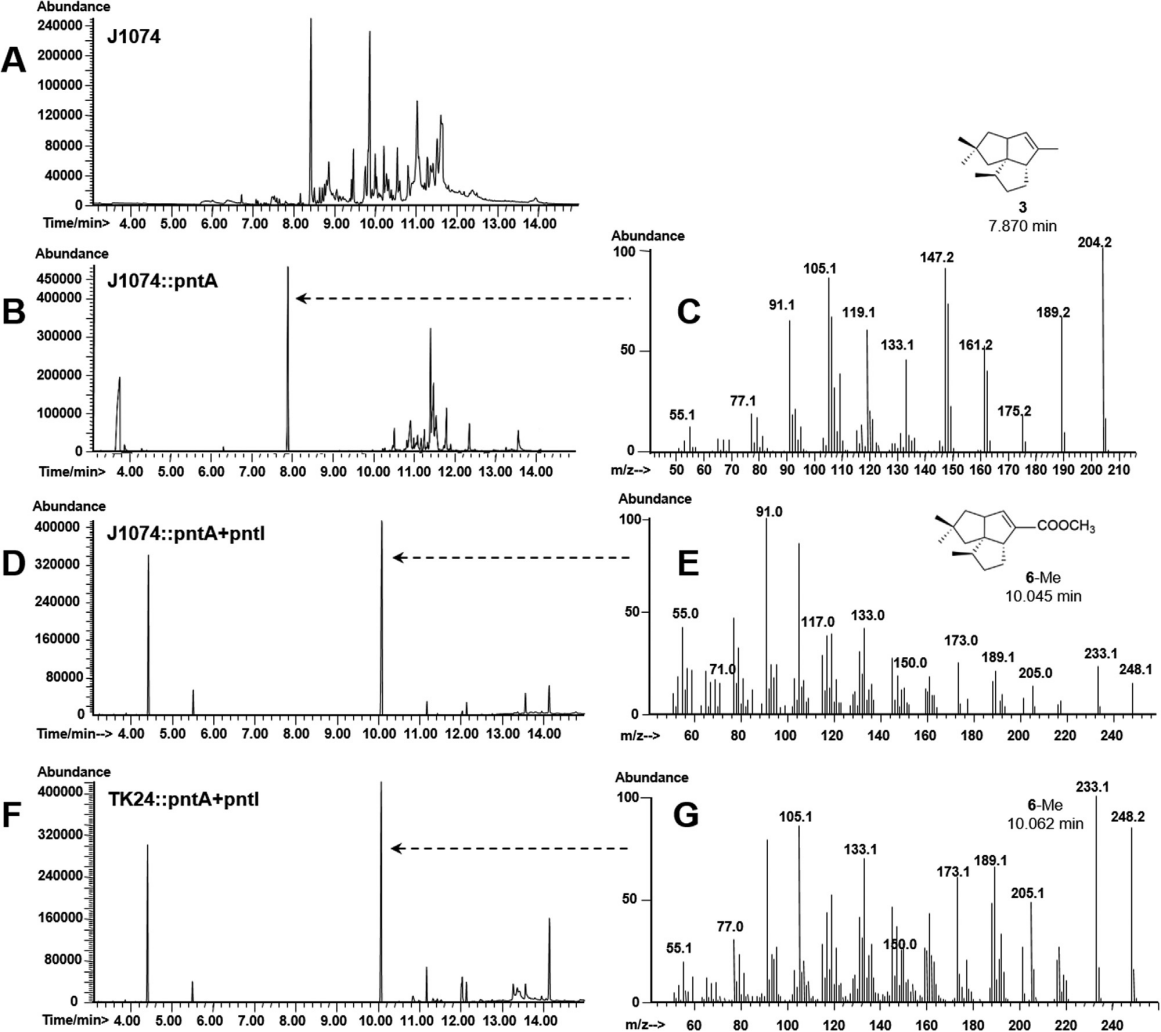
Gas chromatography-mass spectrometry (GC–MS) analysis of *S. albus* and *S. lividans* strains harboring the pentalenene synthase gene *pntA* and the pentalenene oxygenase gene *pntI* from *S. arenae*. A, GC analysis of wild-type *S. albus* J1074; B, GC analysis of the control strain *S. albus* J1074::pWHU1703; C, MS spectra of **3** produced by *S. albus* J1074::pWHU1703; D, GC analysis of *S. albus* J1074::pWHU1702; E, MS spectra of **6**-Me produced by *S. albus* J1074::pWHU1702; F, GC analysis of *S. lividans* TK24::pWHU1702; G, MS spectra of **6**-Me produced by *S. lividans* TK24::pWHU1702.

In a study by Quaderer et al., four pairs of electron transport chain proteins were tested in reactions catalyzed by recombinant PtlI [9]: (1) typical redox pairs from spinach, (2) putidaredoxin and putidaredoxin reductase from *Pseudomonas putida*, (3) flavodoxin (Fld, Sequence ID: WP_001018618) and flavodoxin reductase (Fdr, Sequence ID: WP_000796332.1) from *E. coli*, (4) and ferredoxin FdxD (SAVERM_3129) and ferredoxin reductase FprD (SAVERM_5675) from *S. avermtilis*. Oxidation reactions using the first or second redox pairs were unsuccessful. However, **4** and **5** were detected during incubation with the third or fourth redox pair. Therefore, **6** was not formed, probably due to mismatched redox pairs in the reactions.

The genome of the pentalenolactone producer *S. avermitilis* contains nine ferredoxin (Fdx) genes and six ferredoxin reductase (FDR) genes, whereas the genome of *S. albus*, which was successfully heterologously expressed, contains only four Fdx genes and three FDR genes. To reduce this workload, we screened suitable electron transport chain proteins from *S. albus.* The protein sequences of Fdxs and FDRs from *S. avermitilis* MA-4680 (http://avermitilis.ls.kitasato-u.ac.jp/) were used to search protein databases on *S. albus* (GenBank: CP004370.1), *S. lividans* (GenBank: CP009124.1), and several *Streptomyces* spp. containing the biosynthetic gene cluster for pentalenolactone (**1**), including *S. bingchenggensis* BCW-1 (GenBank: CP002047.1), *S. collinus* TU365 (GenBank: CP006259.1), *S. leeuwenhoekii* C34 (NCBI Reference Sequence: NZ_LN831790.1), *S. cyaneogriseus* NMWT 1 (NCBI Reference Sequence: NZ_CP010849.1), *S. milbemycinicus* SIPI-054 (NCBI Reference Sequence: NZ_CP109672.1), and *S. genisteinicus* CRPJ-33 (NCBI Reference Sequence: NZ_CP060825.1). The results are summarized in Table S4. In the nine Fdx proteins identified, the identity (≥93 %) and similarity (≥96 %) of SAVERM_3129 (FdxD) to its homologs were the highest, followed by SAVERM_6676 (FdxG; identity, ≥81 %; similarity, ≥85 %). In the six predicted FDR proteins (including a predicted ferredoxin-nitrite reductase), SAVERM_5675 (FprD) had the highest levels of identity (≥80 %) and similarity (≥86 %) to its homologs, followed by SAVERM_6097 (FprE; identity, ≥72 %; similarity, ≥80 %). The best matches between two Fdx proteins (SAVERM_3129 and SAVERM_6676) and two FDR proteins (SAVERM_5675 and SAVERM_6097) in *S. albus* were XNR_1673, XNR_5179, XNR_4478, and XNR_4722, which were selected as candidates for further studies.

To avoid interference from the endogenous redox proteins of *Streptomyces* strains, *E. coli* BL21(DE3) (pMH1, pFZ81) [33] was chosen as the host. The DNA fragment carrying the pentalenene synthase gene *penA* was inserted downstream of the T7 promoter of pET21a to generate plasmid pLJ5 (Fig. 4A). Two DNA fragments carrying the pentalenene oxygenase genes *ptlI and pntI* were then inserted downstream of *penA* in pLJ5 to generate the plasmids pLJ80 and pLJ9, respectively (Fig. 4B and C). The three plasmids were transferred into *E. coli* BL21(DE3) (pMH1, pFZ81) respectively, followed by fermentation, isolation, and GC–MS analysis. As expected, the heterologous expression strain of *penA* produced **3** (Fig. 4A). Unexpectedly, **4** and **5** were not detected in the fermentation products of the heterologous expression strains of *penA* and *ptlI* (or *pntI*); however, **6** was detected (Fig. 4B and C). This result differed from those of the *in vitro* biochemical reactions of the recombinant protein PtlI [9]. Based on our previous research, the catalytic activity of an enzyme in a cell is often higher than its activity in an *in vitro* biochemical reaction in the form of a recombinant protein, which may explain why PtlI can catalyze a three-step oxidation in the cell, whereas it can only perform two-step oxidation *in vitro*. Our results confirmed that **6** is the product of the PtlI-catalyzed reaction and that the redox pairs in *E. coli* are functional in the reaction.

**Fig. 4:**
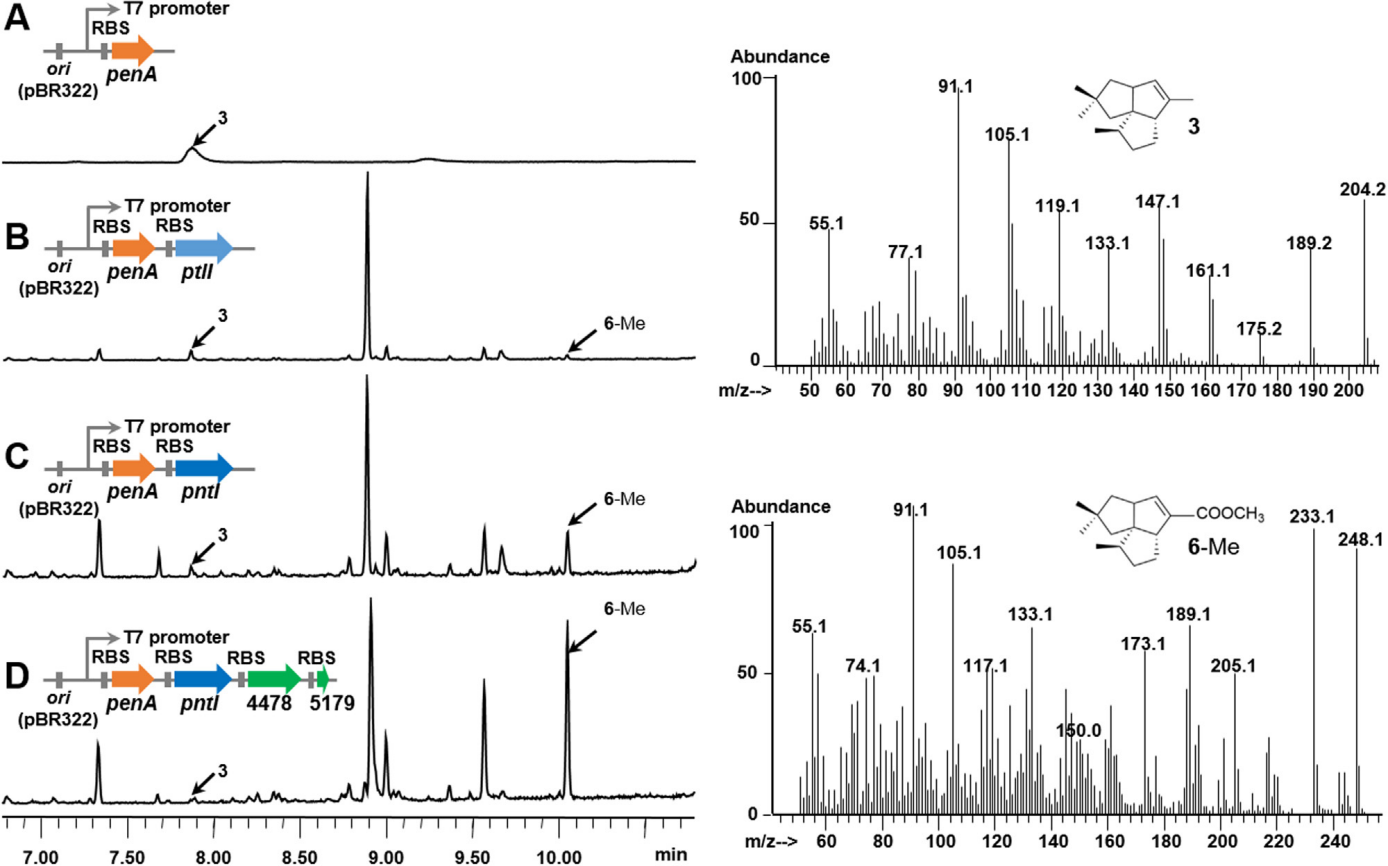
GC–MS analysis of *E. coli* BL21(DE3) strains harboring the pentalenene synthase gene *penA* from *S. exfoliatus* and pentalenene oxygenase gene *pntI* form *S. arenae*. A, BL21(DE3) (pMH1, pFZ81, pLJ5); B, BL21(DE3) (pMH1, pFZ81, pLJ80); C, BL21(DE3) (pMH1, pFZ81, pLJ9); D, BL21(DE3) (pMH1, pFZ81, pLJ62).

It is also worth noting that the catalytic activity of PntI (0.21 mg/L) was significantly higher than that of PtlI (0.08 mg/L), increasing by about 2.6-fold (Figs. 4B and C and 5 [gray columns]). This phenomenon may be caused by differences in enzyme activity due to differences in key amino acids between PntI and PtlI. Both *pntI* and *ptlI* were inserted into a high-copy number vector and were under the control of the strong IPTG-induced promoter T7 and the same RBS so that the transcription levels of the two genes were comparable. However, subtle differences in gene and protein sequences can lead to marked differences in the expression levels and solubility of the final protein, which may be another reason for the differences in their catalytic efficiencies. The catalytic efficiency of Class I P450 enzymes is influenced by the P450: Fdx: FDR ratio. PtlI and PntI were overexpressed in *E. coli* hosts, but the endogenous *E. coli* proteins Fdx and FDR were underexpressed, which may account for the relatively low catalytic efficiency.

**Fig. 5:**
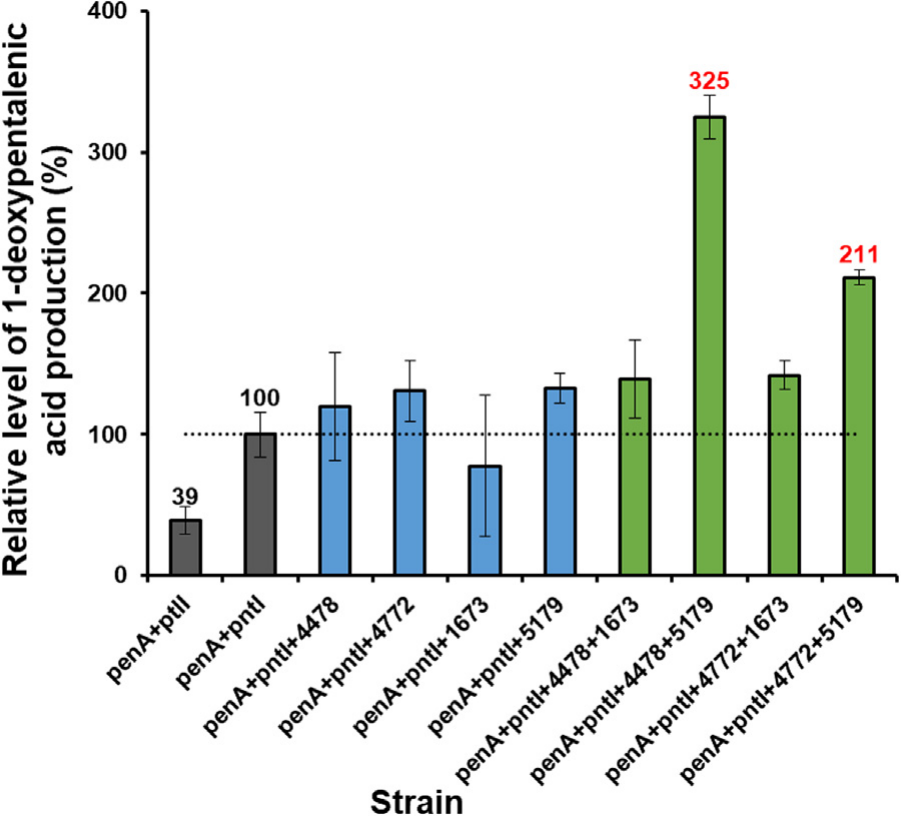
Relative level of 1-deoxypentalenic acid production of *E. coli* BL21(DE3) strains harboring the pentalenene synthase gene penA from S. exfoliatus, the pentalenene oxygenase gene pntI from S. arenae, and electron transport protein genes from S. albus. The relative levels of 1-deocypentalenic acid produced were investigated in the following strains: penA+ptlI, BL21(DE3) (pMH1, pFZ81, pLJ80); penA+pntI, BL21(DE3) (pMH1, pFZ81, pLJ9); penA+pntI+4478, BL21(DE3) (pMH1, pFZ81, pLJ10); penA+pntI+4772, BL21(DE3) (pMH1, pFZ81, pLJ55); penA+pntI+1673, BL21(DE3) (pMH1, pFZ81, pLJ56); penA+pntI+5179, BL21(DE3) (pMH1, pFZ81, pLJ57); penA+pntI+4478+1673, BL21(DE3) (pMH1, pFZ81, pLJ61); penA+pntI+4478+5179, BL21(DE3) (pMH1, pFZ81, pLJ62); penA+pntI+4772+1673, BL21(DE3) (pMH1, pFZ81, pLJ63); and penA+pntI+4772+5179, BL21(DE3) (pMH1, pFZ81, pLJ64). Error bars indicate the standard deviation (*n* = 3).

BL21(DE3) (pMH1, pFZ81, and pLJ9), a strain containing *penA* and *pntI*, was used as the starting strain in the next work. Four Fdx and FDR genes from *S. albus* were inserted downstream of *pntI* in pLJ9 to generate the plasmids pLJ10 (carrying XNR_4478), pLJ55 (carrying XNR_4772), pLJ56 (carrying XNR_1673), and pLJ57 (carrying XNR_5179), which were then transformed into BL21(DE3) (pMH1, pFZ81). Compared to the control strain BL21(DE3) (pMH1, pFZ81, and pLJ9), no significant change in the yield of **6** was observed in the four transformants (Fig. 5, blue columns).

The Fdx and FDR genes were then combined, and four plasmids were constructed: pLJ61 (containing XNR_4478 and XNR_1673), pLJ62 (containing XNR_4478 and XNR_5179), pLJ63 (containing XNR_4772 and XNR_1673), and pLJ64 (containing XNR_4772 and XNR_5179). Compared to the control strain BL21(DE3) (pMH1, pFZ81, pLJ9), the yield of **6** did not change in the two strains harboring Fdx genes XNR_1673, BL21(DE3) (pMH1, pFZ81, pLJ61), and BL21(DE3) (pMH1, pFZ81, pLJ63). In contrast, the yield of **6** increased 2-fold (0.68 mg/L) and 1-fold (0.44 mg/L) in the two strains carrying the Fdx gene XNR_5179, including BL21(DE3) (pMH1, pFZ81, and pLJ62), and BL21(DE3) (pMH1, pFZ81, and pLJ64). (Figs. 4D and 5 [green columns]). These results showed that XNR_5179 and XNR_4478 are the most suitable electron transport proteins for PntI and suggested that Fdxs have a greater effect on the catalytic activity of P450 proteins than FDRs.

To obtain purified proteins for *in vitro* biochemical reactions, we constructed protein expression plasmids for the genes *pntI*, XNR_5179, and XNR_4478, and expressed the recombinant proteins using an *E. coli* protein expression system; however, no soluble proteins were observed. Despite the lack of data on *in vitro* biochemical reactions, we believe that XNR_5179 and XNR_4478 efficiently assist PntI in catalyzing the formation of **6**. Based on the high degree of identity and similarity of their protein amino acid sequences (Table S4), we believe that FdxG (SAVERM_6676) and FprD (SAVERM_5671) in *S. avermitilis* are appropriate natural redox proteins of the pentalenene oxygenase PtlI. In a study by Quaderer [9], **6** was not detected in the pentalenene oxygenase PtlI assay using electron transport proteins from *S. avermitilis* because of the incorrect use of ferredoxins (SAVERM_3129).

The P450 family is diverse and is used to catalyze key steps in drugs such as steroids and antibiotics. In synthetic biology, the combination of P450 with artificial or heterologous redox pairs can extend their substrate range and catalytic capacity. The catalytic process of P450 involves complex electron transfer steps, and its catalytic activity is highly dependent on the efficiency of the electron transfer chain. Studying the fitness of the electron transport chain can help elucidate how electrons are transferred from NAD(P)H to the P450 active site. By optimizing the interactions of relevant proteins in the electron transport chain with P450, the efficiency of electron transfer can be improved, thereby enhancing the catalytic capacity of the enzyme and providing a theoretical basis for protein and metabolic engineering.

Class I cytochrome P450 proteins in bacteria do not fuse with their electron transport chain proteins to form a single protein. In most cases, their genes are not clustered on the chromosome, making the identification of the natural electron transport chain proteins of cytochrome P450 proteins very difficult [[34], [35], [36]]. Early studies on prokaryotic cytochrome P450s used heterologous electron transport chain proteins. However, an increasing number of studies have shown that electron transport chain proteins affect not only enzyme activity but also the chemo-, regio-, and stereoselectivity of the catalytic reaction [37,38]. Therefore, it is necessary to identify the natural intracellular electron transport chain proteins of cytochrome P450 to uncover the true biosynthetic pathways of natural products in a cell.

FR-008/Candicidin, amphotericin, nystatin, and pimaricin are polyene macrolide antibiotics with similar structures. Therefore, FscP, AmphN, NysN, and ScnG/PimG are homologous proteins with a high degree of sequence identity. To investigate the structure-activity relationship of various drugs, the corresponding P450 genes were knocked out, and decarboxylated compounds were derived from the resulting mutant strains to assess alterations in their activity. However, this study did not extend the examination of electron transport chain proteins associated with these P450s. Rosamicin is a macrolide antibiotic, and its carboxylation is achieved by overexpressing the P450 gene *rosC* and electron transport chain protein genes from *P. putida* in an *E. coli* host. However, researchers have not focused on the natural electron transport chain proteins of RosC. Based on the available experimental data, we hypothesized that the concurrent overexpression of *E. coli* electron transport chain proteins in strain BL21(DE3) (pMH1, pFZ81, and pLJ9) (Fig. 4C) could increase the enzymatic activity of P450 pentalenene oxygenase. However, this enhancement was likely to be less than the effect exerted by XNR_5179 and XNR_4478 on enzyme activity.

The heterologous expression of genes *pntI*, Fdx, and FDR in *E. coli* showed specific recognition between P450 and its auxiliary proteins and that only suitable auxiliary proteins can increase the efficiency of P450-catalyzed reactions. This also confirmed that P450 has stringent compatibility requirements for Fdx, with which it interacts directly, whereas the compatibility requirements between Fdx and FDR are relatively less stringent. PntI, XNR_5179, and XNR_4478 from *Streptomyces* were not soluble in *E. coli*; therefore, we believe that it is possible to improve the catalytic activity of PntI by increasing protein solubility through gene codon optimization. Adjusting the organization of genes and replacing the promoter and RBS with those of different strengths will change the ratio of the three proteins in the cell, which may further improve the catalytic activity of PntI.

In conclusion, as shown via gene knockout and heterologous expression in *Streptomyces* spp. and *E. coli*, pentalenene oxygenase can catalyze the production of **6** from **3**, filling the only remaining gap in the biosynthetic pathway of **1**. Hence, FdxG and FprD are considered suitable electron transport chain proteins for pentalenene oxygenases.

## Data Availability Statement

All data generated or analysed during this study are included in this published article and its supplementary information files or are available upon request.

## Declaration of Competing Interest

The authors declare that they have no known competing financial interests or personal relationships that could have appeared to influence the work reported in this paper.

## Appendix Supplementary materials

Image, application 1
