# Supplementary material for: Cytochrome P450-catalyzed allylic oxidation of pentalenene to 1-deoxypentalenic acid in pentalenolactone biosynthesis
Source: Eng Microbiol. 2025 Apr 5;5(2):100206. doi: 10.1016/j.engmic.2025.100206 (PMC12967844; doi:10.1016/j.engmic.2025.100206)
Supplement: Supplementary file 1 [file mmc1.docx]

Supporting Information

Cytochrome P450-Catalyzed Allylic Oxidation of Pentalenene to 1-Deoxypentalenic Acid in Pentalenolactone Biosynthesis

Jing Li ^a,b^, Chengde Zhang ^a^, Shiwen Wu ^a^, Jiao Xue ^a^, Ke Chen ^a^, Zixin Deng ^a^, Dongqing Zhu ^a,^*

^a^ The Key Laboratory of Combinatorial Biosynthesis and Drug Discovery (Ministry of Education), Wuhan University, Wuhan, Hubei Province, 430071, China

^b^ Biomedicine Research Center, the Third Affiliated Hospital of Guangzhou Medical University, Guangzhou, Guangdong Province, 510150, China

**1. Materials and methods**

**2. Table S1-S4 and Figure S1**

**1. Materials and methods**

**1.1. General Materials and Experimental Procedures**

The bacterial strains and plasmids used in this work were listed in Table S1 and S2. Primer sequences are listed in Table S3. Reagents and solvents purchased from Sigma-Aldrich were of the highest quality available. Restriction enzymes, T4 DNA ligase, and DNA polymerase were purchased from New England Biolabs or Yeasen Biotech Co., Ltd. (Wuhan, China) and used according to the manufacturer’s specifications. DNA primers were synthesized by TsingKe Co., Ltd. (Wuhan, China) or Sangon Biotech Co., Ltd. (Shanghai, China). Growth media and conditions used for *E. coli* and *Streptomyces* strains and standard methods for handling *E. coli* and *Streptomyces* in vivo and in vitro were as described previously, unless otherwise noted. All DNA manipulations were performed following standard procedures. DNA sequencing was carried out at TsingKe Co., Ltd. (Wuhan, China) or Sangon Biotech Co., Ltd. (Shanghai, China). GC-MS analyses were carried out on an Agilent 7890A/5975C-GC/MSD at 70 eV electron impact (EI) operating in positive ion mode using an HP5MS capillary column (30 m × 0.25 mm) with a solvent delay of 3 min and a temperature program of 60 °C for 2 min followed by a temperature gradient of 60−280 °C for 11 min at 20 °C/min and a hold at 280 °C for 2 min. The identity of each of the resultant pentalenolactone metabolites was confirmed by direct comparison of retention time and mass spectra with pure authentic standards.

**1.2. Construction of *penI* Mutant *S. exfoliatus* CD2**

The construction of plasmid pWHU1708 used to generate *penI* mutant of *S. exfoliatus* CD2 was listed in Table S2. Plasmid pWHU1708 were transferred into *E. coli* ET12567/pUZ8002, and the unmethylated plasmid was conjugated into *S. exfoliatus* UC5319. Thiostrepton was used to select exconjugants. The single crossover strains were inoculated on SFM plates without antibiotic in order to obtain double crossover strains that had lost thiostrepton resistance. The double crossover strains were confirmed by PCR.

**1.3. Construction of Heterologous Expressional *Streptomyces* strain of *pntI***

The construction of plasmid pWHU1702 harboring *pntA* and *pntI* was listed in Table S2. Plasmid pWHU1702 was transferred into *E. coli* ET12567/pUZ8002, and the unmethylated plasmid was conjugated into *S. albus* J1074 and *S. lividans* TK24 respectively. Apramycin (50 μg/mL) was used to select exconjugants. The exconjugants were confirmed by PCR. PCR amplification of with the primer pair ZCD139F and ZCD139R gave a 2501-bp (or 1014-bp) product from the exconjugant *S. albus* J1074::pWHU1702 and *S. lividans* TK24::pWHU1702. The plasmid pWHU1703 harboring *pentA* was constructed (Table S2) and transferred into *S. albus* J1074 and *S. lividans* TK24 respectively to generate exconjugants. The exconjugants were confirmed by PCR. PCR amplification of with the primer pair ZCD139F and ZCD139R2 gave a 1014-bp product from the exconjugant *S. albus* J1074::pWHU1703 and *S. lividans* TK24::pWHU1703.

**1.4. Analysis of Products from *Streptomyces***

The liquid medium used in fermentation of *S. exfoliatus* contained 0.2% NaCl, 0.5% CaCO_3_, 1% corn gluten meal, 0.1125% bactodextrose, 0.2% blackstrap molasses, and 2% corn starch, pH7.2 [[1](#_ENREF_1)]. The liquid medium used in fermentation of *S. albus* and *S. lividans* contained 2% soybean powder and 2% mannitol, pH 7.2. After incubation at 30 ℃ for 6 days, the culture was acidified to pH 2.4 with H_2_SO_4_ and extracted with chloroform. The organic layer was dried over anhydrous Na_2_SO_4_, concentrated, methylated with TMS-CHN_2_, and analyzed by GC-MS.

**1.5 Analysis of Products from *E. coli***

The plasmids harboring pentalenene synthase genes, pentalenene oxygenase genes and redox proteins genes were constructed (Table S2) and transferred into *E. coli* BL21(DE3). The resulting *E. coli* strains were cultured overnight at 37 °C in LB medium with antibiotics. Then, 1% (v/v) of the culture was transferred to fresh 2×YT medium and incubated at 37 °C until the OD600 reached 0.6−0.8. IPTG was added to a final concentration of 0.15 mM, and the culture was further incubated at 28 °C for 4 h. The culture was acidified to pH 2.4 with H_2_SO_4_ and extracted with chloroform. The organic layer was dried over anhydrous Na_2_SO_4_, concentrated, methylated with TMS-CHN_2_, and analyzed by GC-MS.

**2. Table S1-S4 and Figure S1**

Table S1**.** Bacterial strains used in this study

| Strain | Relevant phenotype and/or characteristics | Source or reference |
| --- | --- | --- |
| *Streptomyces* strains |  |  |
| *S. exfoliatus* UC5319 | Wild-type strain, pentalenolactone producer | Upjohn Co. (Pfizer) |
| *S. exfoliatus* CD2 | *ΔpntI* in-frame deletion mutant | This work |
| *S. arenae* TU469 | Wild-type strain, pentalenolactone producer | DSM 40734, DMSZ |
| *S. avermitilis* NRRL 8165 | Wild-type strain, neopentalenolactone producer | NRRL |
| *S. albus* J1074 | Wild-type strain |  |
| *S. lividans* TK24 | Wild-type strain |  |
| *Escherichia coli* strains |  |  |
| DH10B | F^-^ *mcrA, Δ(mrr-hsdRMS-mcrBC),* φ80d*lacZΔM15, ΔlacX74, recA1, endA1, araD139, Δ(ara, leu)7697, galU, galK, rpsL, nupG* | Gibco BRL |
| ET12567/pUZ8002 | F^–^ *ara-14, leuB6, fhuA13, lacY1, tsx-78, supE44, glnV44, galK2, galT22, mcrA, dcm-6, hisG4, rfbD1, rpsL136, dam-13::*Tn*9, xyl-5, mtl-1, recF143, thi-1, mcrB, hsdR2, hsdS::*Tn*10* pUZ8002*:* (derivative of pUB307, *tra*) | [[2](#_ENREF_2), [3](#_ENREF_3)] |
| BL21(DE3) | F–, *ompT*, *hsdSB* (rB- mB-) *gal*, *dcm* (DE3) | Invitrogen |

Table S2. Plasmids used in this study

| Plasmid | Relevant phenotype and/or characteristics | Source or reference |
| --- | --- | --- |
| pJTU1278 | pIJ101 derivative, *bla*, *tsr*, *oriT*, *sti* | [[4](#_ENREF_4)] |
| pIB139 | *aac(3)IV*, *oriT*, *int*, *ermE*p*** | [[5](#_ENREF_5)] |
| pFZ81 | P*lac*: ERG12, ERG8, MVD1 and N-terminal His-tagged Idi, *ori*: pBBR1MCS *ori* | [[6](#_ENREF_6)] |
| pMH1 | P*lac*: AtoB, ERG13 and N-terminal His-tagged tHMG1, *ori*: p15A | [[6](#_ENREF_6)] |
| pClone 007-Blunt | *bla*, T7 promoter | Tsingke |
| pET21a | *bla*, orif1, lacI, oripBR322, T7 promoter | Novagen |
| pET26b | KanR, *ori*^f1^, *lacI*, *ori*^pBR322^, T7 promoter, *pelB* | Novagen |
| pET28a | KanR, *ori*^f1^, *lacI*, *ori*^pBR322^, T7 promoter | Novagen |
| pWHU1607 | 1370-bp BamHI + XhoI DNA fragment carrying *ptlI* amplified from *S*. *avermitilis* NRRL 8165 by using primer pair CK4F and CK4R, inserted into the corresponding site of pET28a | This work |
| pWHU1702 | **pIB139-*pntA-pntI*:** 2502-bp NdeI + NotI DNA fragment carrying *pntA* and *pntI* amplified from *S. arenae* TU469 by using primer pair ZCD139F and ZCD139R, inserted into the corresponding site of pIB139 | This work |
| pWHU1703 | **pIB139-*pntA:*** 1014-bp NdeI + NotI DNA fragment carrying *pntA* amplified from *S. arenae* TU469 by using primer pair ZCD139F and ZCD139R2, inserted into the corresponding site of pIB139 | This work |
| pWHU1708 | 1598-bp XbaI + NdeI DNA fragment carrying partial *penB, penA* and partial *penI* , and 1499-bp NdeI + BamHI DNA fragment carrying partial *penI* and downstream of *pen* gene cluster amplified from *S. exfoliatus* UC5319 by using primer pair UC1F and UC1R, and primer pair UC2F and UC2R, inserted into the XbaI + BamHI site of pJTU1278 | This work |
| pLJ1 | 1014-bp DNA fragment carrying *penA* amplified from *S. exfoliatus* UC5319 by using primer pair LJ1F and LJ1R, inserted into pClone 007-Blunt | This work |
| pLJ2 | 1398-bp DNA fragment carrying *pntI* amplified from *S. arenae* TU469 by using primer pair LJ2F and LJ2R, inserted into pClone 007-Blunt | This work |
| pLJ3 | 1287-bp DNA fragment carrying XNR_4478 amplified from *S. albus* J1074 by using primer pair LJ3F and LJ3R, inserted into pClone 007-Blunt | This work |
| pLJ5 | **pET21a-*penA*:** 1026-bp BamHI + XhoI DNA fragment carrying *penA* from pLJ1, inserted into the corresponding site of pET21a | This work |
| pLJ6 | 1386-bp BamHI + XhoI DNA fragment carrying *pntI* from pLJ2, inserted into the corresponding site of pET28a | This work |
| pLJ7 | 1275-bp BamHI + XhoI DNA fragment carrying XNR_4478 from pLJ3, inserted into the corresponding site of pET28a | This work |
| pLJ9 | **pET21a-*penA-pntI*:** 1523-bp XbaI + XhoI DNA fragment carrying *pntI* from pLJ6, inserted into SpeI + XhoI site of pLJ5 | This work |
| pLJ10 | **pET21a-*penA-pntI-4478*:** 1412-bp XbaI + XhoI DNA fragment carrying XNR_4478 from pLJ7, inserted into SpeI + XhoI site of pLJ9 | This work |
| pLJ52 | 1212-bp BamHI + XhoI DNA fragment carrying XNR_4772 amplified from *S. albus* J1074 by using primer pair LJ41F and LJ41R, inserted into the corresponding site of pET28a | This work |
| pLJ53 | 343-bp NdeI + XhoI DNA fragment carrying XNR_1673 amplified from *S. albus* J1074 by using primer pair LJ42F and LJ42R, inserted into the corresponding site of pET26b | This work |
| pLJ54 | 331-bp NdeI + XhoI DNA fragment carrying XNR_5179 amplified from *S. albus* J1074 by using primer pair LJ43F and LJ43R, inserted into the corresponding site of pET26b | This work |
| pLJ55 | **pET21a-*penA-pntI-4772*:** 1337-bp XbaI + XhoI DNA fragment carrying XNR_4772 from pLJ52, inserted into SpeI + XhoI site of pLJ9 | This work |
| pLJ56 | **pET21a-*penA-pntI-1673*:** 372-bp XbaI + XhoI DNA fragment carrying XNR_1673 from pLJ53, inserted into SpeI + XhoI site of pLJ9 | This work |
| pLJ57 | **pET21a-*penA-pntI-5179*:** 354-bp XbaI + XhoI DNA fragment carrying XNR_5179 from pLJ54, inserted into SpeI + XhoI site of pLJ9 | This work |
| pLJ61 | **pET21a-*penA-pntI-4478-1673*:** 372-bp XbaI + XhoI DNA fragment carrying XNR_1673 from pLJ53, inserted into SpeI + XhoI site of pLJ10 | This work |
| pLJ62 | **pET21a-*penA-pntI-4478-5179*:** 354-bp XbaI + XhoI DNA fragment carrying XNR_5179 from pLJ54, inserted into SpeI + XhoI site of pLJ10 | This work |
| pLJ63 | **pET21a-*penA-pntI-4772-1673*:** 372-bp XbaI + XhoI DNA fragment carrying XNR_1673 from pLJ53, inserted into SpeI + XhoI site of pLJ55 | This work |
| pLJ64 | **pET21a-*penA-pntI-4772-5179*:** 354-bp XbaI + XhoI DNA fragment carrying XNR_5179 from pLJ54, inserted into SpeI + XhoI site of pLJ55 | This work |
| pLJ80 | **pET21a-*penA-ptlI*:** 1451-bp XbaI + XhoI DNA fragment carrying *ptlI* from pWHU1607, inserted into SpeI + XhoI site of pLJ5 | This work |

Table S3 Primers used in this study

| Primer | Sequence (5’-3’), (restriction enzyme site underlined) | Purpose |
| --- | --- | --- |
| UCF | GGAGGCCCGCGGCGAGCAGA | CD2 |
| UCR | TCATGTGAGGTCACCACCGG | CD2 |
| UC1F | GCTCTAGACCAGCTCGTCGACGATGTTCTC (XbaI) | pWHU1708 |
| UC1R | GGAATTCCATATGGGTCATGAAGTTCACTG (NdeI) | pWHU1708 |
| UC2F | GGAATTCCATATGGTCTACCACCCACGCCG (NdeI) | pWHU1708 |
| UC2R | CGCGGATCCCCGTCCGCCCACGACAGTGCA (BamHI) | pWHU1708 |
| ZCD139F | GGAATTCCATATGATGCCCCAGGACGTCGACTTCC (NdeI) | pWHU1702 |
| ZCD139R | ATAAGAATGCGGCCGCTCACCGGGTGAGGCGGGA (NotI) | pWHU1702 |
| ZCD139R2 | ATAAGAATGCGGCCGCCTAGTGGGTGATCCGGCC (NotI) | pWHU1703 |
| CK4F | GGGAATTCCATATGTCCCAGCACACCTTC (NdeI) | pWHU1607 |
| CK4R | CCGCTCGAGCTACTGACCCGGCGTACG (XhoI) | pWHU1607 |
| LJ1F | CCGGGATCCATGCCCCAGGACGTCGACTTC (BamHI) | pLJ1 |
| LJ1R | GGCCTCGAGACTAGTCTAGTGGGCGCTGCT (SpeI+XhoI) | pLJ1 |
| LJ2F | CCGGGATCCATGACCGAGCAGACCACCTTC (BamHI) | pLJ2 |
| LJ2R | GGCCTCGAGACTAGTTCACCGGGTGAGGCG (SpeI+XhoI) | pLJ2 |
| LJ3F | CCGGGATCCGTGGTCGACGCGGATCAGACA (BamHI) | pLJ1 |
| LJ3R | GGCCTCGAGACTAGTTCAGGCCCCGAGGCT (SpeI+XhoI) | pLJ1 |
| LJ41F | CGCGGATCCGTGGTGGTCGTCGGCGCGGG (BamHI) | pLJ52 |
| LJ41R | GGCCTCGAGACTAGTTCAGGCCGTGGTCGA (SpeI+XhoI) | pLJ52 |
| LJ42F | GGAATTCCATATGGTGACCTACGTCATCGCG (NdeI) | pLJ53 |
| LJ42R | GGCCTCGAGACTAGTCTACTGGTTCTGCGGCGGCA (SpeI+XhoI) | pLJ53 |
| LJ43F | GGAATTCCATATGGTGCAGGAGGAGGCCGGG (NdeI) | pLJ54 |
| LJ43R | GGCCTCGAGACTAGTCTACGACGCCTCGGGTCCGT (SpeI+XhoI) | pLJ54 |

Table S4 Comparisons of predicted ferredoxin and ferredoxin proteins of *Streptomyces* strains

| ***S. avermitilis* MA-4680** | | **Locus_tag (aa*, % Identity/ Similarity**)** | | | | | | | |
| --- | --- | --- | --- | --- | --- | --- | --- | --- | --- |
| **Name** | **SAVERM_(aa*)** | **SBI_** | **B446_** | **BN2145_RS** | **TU94_RS** | **NLG24_RS** | **IAG43_RS** | **SLIV_** | **XNR_** |
| **Ferredoxin** |  |  |  |  |  |  |  |  |  |
| **PteE/FdxI** | **411 (64)** | 00377 (75, 36/49) | 32520 (69, 62/73) | 06390 (63, 56/63) | 03640 (64, 40/54) | 03060 (63, 54/71) | 05595 (103, 48/58) | 34065 (66, 57/66) | 5864 (64, 41/57) |
| **FdxA** | **582 (69)** | 00377 (75, 65/80) | 13760 (79, 63/79) | 09055 (65, 43/63) | 03640 (64, 41/53) | 48745 (75, 65/80) | None | 18930 (70, 38/46) | 5705 (66, 40/53) |
| **FdxB** | **1610 (65)** | 00425 (99, 58/76) | 32520 (69, 42/59) | 32595 (64, 72/84) | 03640 (64, 71/81) | 48495 (75, 58/76 ) | None | 00920 (73, 48/65) | 5705 (66, 58/75) |
| **FdxC** | **2296 (108)** | 04094 (108, 68/83) | 27895 (109, 79/85) | 14450 (106, 71/84) | 21270 (106, 71/84) | 19075 (108, 68/83) | 25615 (108, 84/90) | 12705 (106, 70/83) | 1673 (106, 69/82) |
| **FdxD** | **3129 (106)** | 04094 (108, 94/96) | 24135 (106, 94/96) | 14450 (106, 93/96) | 21270 (106, 93/96) | 19075 (108, 94/96) | 20950 (106, 96/99) | 12705 (106, 96/98) | 1673 (106, 96/97) |
| **FdxE** | **4856 (75)** | 06108 (203, 33/45) | 24135 (106, 41/53) | None | None | None | 18400 (75, 95/96) | None | 1673 (106, 41/53) |
| **FdxF** | **5853 (77)** | 07594 (76, 69/76) | 12170 (77, 73/80) | None | 13090 (70, 34/49) | 36910 (76, 69/76) | 32035 (62, 35/55) | None | 5864 (64, 29/48) |
| **FdxG** | **6676 (101)** | 08477 (98, 81/85) | 08515 (100, 87/89) | 29045 (101, 88/93) | 06890 (101, 88/92) | 41395 (96, 86/90) | 05595 (103, 84/87) | 29500 (99, 89/92) | 5179 (100, 88/91) |
| **FdxH** | **7470 (94)** | 00654 (63, 52/58) | 32520 (69, 78/89) | 06390 (63, 69/74) | 03640 (64, 44/57) | 03060 (63, 52/58) | 05595 (103, 40/50) | 34065 (66, 80/86) | 5864 (64, 42/54) |
| **Ferredoxin reductase** |  |  |  |  |  |  |  |  |  |
| **FprA** | **583 (454)** | 05210 (476, 66/76) | 06685 (458, 63/74) | 09050 (416, 47/57) | 25630 (417, 38/50) | 26325 (476, 66/76) | 07920 (412, 35/49) | 03690 (420, 38/51) | 4772 (395, 36/51) |
| **FprB** | **1507 (448)** | 02050 (448, 73/78) | 05650 (446, 80/83) | 31770 (464, 81/85) | 04170 (464, 81/85) | 08700 (448, 73/78) | 09925 (455, 41/54) | 34535 (454, 40/55) | 2490 (535, 46/56) |
| **FprC** | **1609 (405)** | 00421 (403, 52/66) | 13765 (464, 44/59) | 09650 (417, 55/62) | 25630 (417, 54/62) | 48515 (403, 52/66) | 09750 (420, 40/56) | 03690 (420, 39/55) | 4772 (395, 41/54) |
| **FprD** | **5675 (421)** | 07356 (426, 80/86) | 12720 (421, 89/94) | 25370 (421, 89/93) | 10615 (421, 87/94) | 35730 (426, 80/86) | 09750 (420, 85/90) | 25340 (421, 88/92) | 4478 (420, 83/89) |
| **FprE** | **6097 (421)** | 07856 (399, 74/81) | 11055 (395, 83/88) | 26855 (387, 83/90) | 09090 (387, 83/89) | 38270 (416, 72/80) | 07920 (412, 74/80) | 27175 (407, 82/88) | 4772 (395, 71/77) |
| **FprF** | **6956 (413)** | 00366 (390, 53/62) | 11055 (395, 40/51) | 26855 (387, 40/50) | 09090 (387, 40/50) | 48800 (393, 54/63) | 07920 (412, 39/50) | 27175 (407, 41/51) | 4772 (395, 40/49) |

* aa, amino acid;

** % Identity/ Similarity, Percentage of identity and similarity of the amino acid sequence to the corresponding protein in *S. avermitilis* MA-4680;

SAVERM_, *S. avermitilis* MA-4680, <http://avermitilis.ls.kitasato-u.ac.jp/cyp.html>

SBI_, *S. bingchenggensis* BCW-1, <https://www.ncbi.nlm.nih.gov/nuccore/CP002047.1>

B446_, *S. collinus* TU365, <https://www.ncbi.nlm.nih.gov/nuccore/529195098>

BN2145_RS, *S. leeuwenhoekii* C34, <https://www.ncbi.nlm.nih.gov/nuccore/NZ_LN831790.1>

TU94_RS, *S. cyaneogriseus* NMWT 1, <https://www.ncbi.nlm.nih.gov/nuccore/764487836>

NLG24_RS, *S. milbemycinicus* SIPI-054, <https://www.ncbi.nlm.nih.gov/nuccore/2323612663>

IAG43_RS, *S. genisteinicus* CRPJ-33, <https://www.ncbi.nlm.nih.gov/nuccore/NZ_CP060825.1>

SLIV_, *S. lividans* TK24, <https://www.ncbi.nlm.nih.gov/nuccore/CP009124>

XNR_, *S. albus* J1074, <https://www.ncbi.nlm.nih.gov/nuccore/CP004370>


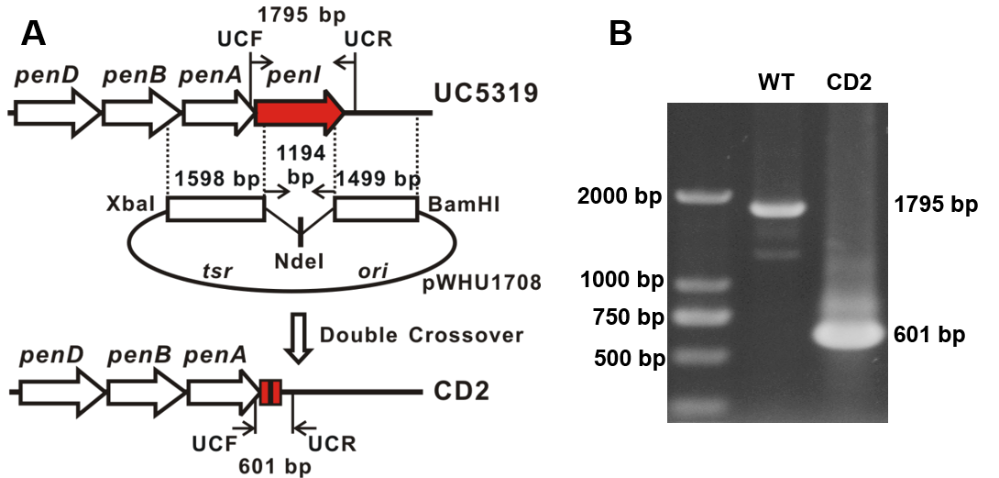


Fig. S1. Construction of the Δ*penI* mutant strain CD2. (A) Schematic representation of the deletion of *penI*. (B) Confirmation of the mutant by PCR.

**Reference**

[1] M.J. Seo, D.Q. Zhu, S. Endo, H. Ikeda, D.E. Cane, Genome Mining in Streptomyces. Elucidation of the Role of Baeyer-Villiger Monooxygenases and Non-Heme Iron-Dependent Dehydrogenase/Oxygenases in the Final Steps of the Biosynthesis of Pentalenolactone and Neopentalenolactone, Biochemistry-Us 50 (2011) 1739-1754, doi: 10.1021/bi1019786.

[2] M.S.B. Paget, L. Chamberlin, A. Atrih, S.J. Foster, M.J. Buttner, Evidence that the extracytoplasmic function sigma factor σ sigmaE is required for normal cell wall structure in *Streptomyces coelicolor* A3(2), J Bacteriol 181 (1999) 204-211, doi: Doi 10.1128/Jb.181.1.204-211.1999.

[3] D.J. Macneil, K.M. Gewain, C.L. Ruby, G. Dezeny, P.H. Gibbons, T. Macneil, Analysis of *Streptomyces*-Avermitilis Genes Required for Avermectin Biosynthesis Utilizing a Novel Integration Vector, Gene 111 (1992) 61-68, doi: Doi 10.1016/0378-1119(92)90603-M.

[4] Y.L. He, Z.J. Wang, L.Q. Bai, J.D. Liang, X.F. Zhou, Z.X. Deng, Two pHZ1358 Derivative Vectors for Efficient Gene Knockout in Streptomyces, J Microbiol Biotechn 20 (2010) 678-682, doi: 10.4014/jmb.0910.10031.

[5] C.J. Wilkinson, Z.A. Hughes-Thomas, C.J. Martin, I. Böhm, T. Mironenko, M. Deacon, M. Wheatcroft, G. Wirtz, J. Staunton, P.F. Leadlay, Increasing the efficiency of heterologous promoters in actinomycetes, J Mol Microb Biotech 4 (2002) 417-426.

[6] F.Y. Zhu, X.F. Zhong, M.Z. Hu, L. Lu, Z.X. Deng, T.G. Liu, In vitro reconstitution of mevalonate pathway and targeted engineering of farnesene overproduction in Escherichia coli, Biotechnol Bioeng 111 (2014) 1396-1405, doi: 10.1002/bit.25198.
